# GRECCS AS A NETWORK TO ENHANCE THE VA PRIMARY CARE WORKFORCE

**DOI:** 10.1093/geroni/igz038.2813

**Published:** 2019-11-08

**Authors:** Josea Kramer, Marianne Shaughnessy

**Affiliations:** 1 VA Greater Los Angeles Healthcare System GRECC, Los Angeles, California, United States; 2 Veterans Health Administration Geriatrics and Extended Care Services, Office Geriatrics Programs, Washington, District of Columbia, United States

## Abstract

The VA Geriatric Scholars Program is a workforce development program to integrate geriatrics into primary care in all VA clinics. The program has increased career satisfaction among clinicians, improved quality of care for aging Veterans, changed provider prescribing behaviors and improved patient outcomes. The core curriculum is an intensive course in geriatrics, an intensive workshop in quality improvement (QI) and a micro QI project to demonstrate application of new knowledge to local needs of patients and clinic processes; this is a longitudinal program and alumni continue to develop skills over time through a series of elective activities that tailor learning to self-identified gaps in career development and training. The program is a collaboration among the Geriatric Research Education and Clinical Centers (GRECC), centers of excellence that have been leveraged to create a network of expertise and creativity to develop the core and elective educational activities. This symposium focuses on recent outcomes of three educational components that improve quality of care for older Veterans. The Palo Alto GRECC developed an intensive course in gerontological psychology to enhance the skills and competencies of psychologists leading to greater job effectiveness. The Bronx GRECC developed an interdisciplinary training program for all clinical and non-clinical staff in rural clinics, leading to improved recognition of functional and safety concerns among older veterans. The Tennessee Valley GRECC developed the core QI component and many of these micro-QI projects have been sustained, spread or recognized by VA as Promising Practices.

